# Developing Self-Awareness in Robots via Inner Speech

**DOI:** 10.3389/frobt.2020.00016

**Published:** 2020-02-19

**Authors:** Antonio Chella, Arianna Pipitone, Alain Morin, Famira Racy

**Affiliations:** ^1^RoboticsLab, Dipartimento di Ingegneria, Università degli Studi di Palermo, Palermo, Italy; ^2^Istituto di Calcolo e Reti ad Alte Prestazioni (ICAR), Consiglio Nazionale delle Ricerche, Palermo, Italy; ^3^Department of Psychology, Mount Royal University, Calgary, AB, Canada; ^4^Researcher, Mount Royal University, Calgary, AB, Canada

**Keywords:** inner speech, self-awareness, robot, human-robot interaction, cognitive cycle

## Abstract

The experience of inner speech is a common one. Such a dialogue accompanies the introspection of mental life and fulfills essential roles in human behavior, such as self-restructuring, self-regulation, and re-focusing on attentional resources. Although the underpinning of inner speech is mostly investigated in psychological and philosophical fields, the research in robotics generally does not address such a form of self-aware behavior. Existing models of inner speech inspire computational tools to provide a robot with this form of self-awareness. Here, the widespread psychological models of inner speech are reviewed, and a cognitive architecture for a robot implementing such a capability is outlined in a simplified setup.

## Introduction

The idea of implementing self-awareness in robots has been popular in science-fiction literature and movies for a long time. This quest is also becoming increasingly prevalent in scientific research, with articles, special topics, books, workshops, and conferences dedicated to it.

It is widely assumed that there are two dimensions of awareness (see Dehaene et al., 2017), and namely, awareness as experience and awareness as self-monitoring, i.e., self-awareness. In essence, awareness as experience occurs when an agent perceives the environment and experiences it from within in the form of images, sensations, thoughts, and so on (see Block et al., 2019); as such, awareness (or consciousness) exists when an organism can focus attention outward toward the environment (Duval and Wicklund, 1972). Instead, self-awareness takes place when the agent focuses attention inward and apprehends the self in its diverse manifestations, like emotions, thoughts, attitudes, sensations, motives, physical attributes, which frequently involves a verbal narration of inner experiences (Morin, 2011).

Models of awareness and self-awareness are being proposed, each with idiosyncratic views of what the aforementioned concepts constitute, as well as different suggestions on how to implement them in artificial agents (see among others, Tononi and Edelman, 1998; Gray et al., 2007; Seth, 2010; Edlund et al., 2011; Oizumi et al., 2014; Tononi et al., 2016; Juel et al., 2019). For reviews, see Reggia (2013) and Chella et al. (2019).

The proposed approach focuses on implementing a form of robot self-awareness by developing inner speech in the robot. Inner speech is known to importantly participate in the development and maintenance of human self-awareness (Morin, 2018); thus, self-talk in robots is an essential behavioral capability of robot self-awareness.

More in detail, the paper discusses a computational model of inner speech. The proposed model is based on the cognitive architecture described by Laird et al. (2017). Therefore, the approach is based on the complex interplay of different blocks as a shape classifier, a speech recognition, and a speech production system, a Short-Term memory, a procedural, a declarative Long-Term Memory, and more. Preliminary versions of the architecture are presented in Chella and Pipitone (2019, 2020).

To the best of the authors' knowledge, inner speech has not been taken into account in studies concerning human-robot interactions. According to the triadic model of trust in human-robot interactions (Hancock et al., 2011), inner speech (and/or out loud self-directed speech—private speech) would enhance trust in human-robot cooperation by strengthening the anthropomorphism of the robot itself. A robot aimed with inner speech would be more able to perform self-disclosure, and to establish social interactions (Cassell and Bickmore, 2003). Transparency in the interactions with human teammates would be enhanced too (Lee and See, 2004; Hoff and Bashir, 2015).

The need for explorations in the relationships between robot self-awareness, and human-robot trust has been claimed by Mittu et al. (2016). On the same line, Abbass et al. (2018) discuss the definition of trusted autonomy in robots to include the “awareness of self.”

In what follows, we outline a definition of human self-awareness and various self-related phenomena from a psychological standpoint, and offer explanations as to why implementing these attributes in robots would be beneficial. In short, a robot with forms of self-awareness should be able to increase the social competencies of the robot itself by making the robot more acceptable and trustworthy in the social context. The robot's inner speech may be audible, and thus the cognitive cycle may be transparent to the user, in the sense that the user may easily follow the cognitive cycle of the robot and assign the correct level of trust in the robot operations.

We present existing approaches to self-awareness deployment in robots, observing that the crucial potential role of inner speech is only marginally addressed. This motivates our proposal, which, to be fully appreciated, requires a general survey oriented to the robotics and AI community, of existing information about inner speech in humans, with an emphasis on how it relates to self-awareness. This section is followed by the presentation of a novel and detailed cognitive architecture model designed to instigate inner speech in robots. The cognitive architecture model heavily rests on an interactive cycle between perception (e.g., proprioception), action (e.g., covert articulation), and memory (short-term and long-term memory). We also discuss additional components of self-awareness (Morin and Racy, in press)—beyond inner speech—that should eventually be developed in robots to reach full-blown self-awareness, such as social comparison and future-oriented thinking. We conclude with some proposals regarding possible ways of testing self-awareness in humanoid robots.

## Self-Awareness

### What Self-Awareness Entails

From a psychological point of view, “self-awareness” represents the ability to become the object of one's attention (Duval and Wicklund, 1972). It constitutes the active state of individuating, processing, storing, retrieving information about the self (Morin, 2011). Synonyms include “self-observation,” “introspection,” and “self-focused attention.” Self-aspects comprise private (unobservable) components such as thoughts, emotions, and motives, as well as public (visible) components as appearance, mannerisms, and others' opinion of self (Davies, 2005; for a detailed list see Morin, 2006, **Figure 2**).

Critical individual differences exist in terms of self-awareness, the natural disposition to focus more or less frequently on the self (Fenigstein et al., 1975). To illustrate, some people more often focus on private self-aspects than public ones, predisposing them to introversion and social awkwardness.

Trapnell and Campbell (1999) introduced an essential difference between “self-reflection” and “self-rumination.” The former entails a non-anxious, healthy type of self-attention generally linked to positive outcomes (e.g., self-regulation and self-knowledge; also see Silvia and O'Brien, 2004), while the latter, an anxious, unhealthy, repetitive form of self-focus about negative aspects of self, associated with dysfunctional outcomes (e.g., anxiety, depression; Mor and Winquist, 2002). Joireman et al. (2002) used the term “self-absorption” to designate the state of self-rumination. It is unclear why self-focused attention can often take a wrong turn and become self-rumination. The type of self-awareness one wants to implement in robots ought to be reflective—not ruminative. Thus, it is crucial to ensure that potential rumination gets disabled as soon as it starts occurring if it does.

The forms mentioned above of self-awareness are measured with self-report questionnaires, frequency of first-person pronouns use, and self-description tasks; they can also be induced by the exposition of participants to self-focusing stimuli as cameras, mirrors, and audiences (Carver and Scheier, 1978). (For measurements and manipulations of self-awareness see also Morin, 2011 Table 2).

The above arguments are essential for a cognitive architecture for a social robot because any artificial intelligence that successfully interacts with humans should need to be able to use first-person pronouns, self-describe, and be responsive to self-focusing stimuli in its surrounding environment.

The term “metacognition,” a specific case of self-reflection, is used to designate an awareness of one's thoughts (Smith, 2009). The term “insight” concerns the ability to identify and express one's emotions (Grant et al., 2002), while the term “agency” refers to a feeling that one is causally responsible for one's actions (Kelso, 2016). The terms “self-distancing,” and “self-immersion” represent different opposite forms of self-reflection, where the former consists in examining the self from some distance, and the latter, with no distance (Kross and Ayduk, 2017). Self-immersion and self-distancing can be experimentally manipulated by asking participants to talk to themselves by using first-person pronouns (e.g., “me”; self-immersion), or by using their name (“John”; self-distancing; Zell et al., 2012). Robots that humans can relate to should ultimately be able to demonstrate at least some simple form of the above self-reflective processes.

The use of personal pronouns, self-conscious emotions, mirror self-recognition, and pretend play, all emerge between the ages of 15 and 24 months in humans, probably because of the parallel development of self-reflection (Lewis and Ramsay, 2004). Self-aware emotions like pride, shame, envy, embarrassment, and guilt begin during the second year of life (Buss, 1980). Rochat (2003) proposed five developmental stages of self-awareness: (1) *Differentiation* (from birth) takes place when the infants physically differentiate self from non-self; (2) *Situation* (2 months) occurs when the infants situate themselves in relation to other persons; (3) *Identification* (2 years) emerges when children, become capable of self-recognition when they are in front of a mirror; (4) *Permanence* (3 years) is when children know that their feeling of self is persevering across space and time; (5) ultimately, self-consciousness (*meta self-awareness*; 4–5 years) is considered to be present when children perceive themselves as seen by others. A self-aware AI agent should be able to apprehend itself across time and space, as well as say things like “Hi, my name is Adam, my birthday is next week, and I am 5 years old.”

Multiple brain areas typically increase in activation during self-reflection tasks such as autobiography (past-oriented thoughts), prospection (future-oriented thoughts), emotions, agency, Theory-of-Mind (thinking about others' mental states), and preferences (see Morin and Hamper, 2012). Increased activation occurs during these tasks in the medial prefrontal cortex, inferior parietal lobules, posterior cingulate/precuneus, and regions of the medial and lateral temporal lobes (Denny et al., 2012), more so on the left part of the brain (Morin, 2010). Increased activation of these regions is also associated with the ‘resting state' when participants are invited to close their eyes and do nothing (Buckner et al., 2008). This suggests that the people in a resting state are really not resting but instead thinking about an array of self-related topics such as remembering a past event and imagining some future one; simply put, they are in a state of self-awareness (Davey et al., 2016).

### Why Would Self-Awareness Benefit Robots?

From the above review of the psychological literature, it appears that self-awareness represents a part of an adaptation strategy for navigating the environment, social world, and self, increasing the likelihood of survival. Carruthers et al. (2012) note that “… organisms evolve a capacity for self-knowledge in order better to manage and control their own mental lives. By being aware of some of their mental states and processes, organisms can become more efficient and reliable cognizers and can make better and more adaptive decisions as a result” (pp. 14–15).

From the AI perspective, a robot with some form of self-awareness will better self-adapt to unforeseen environmental changes by engaging in the form of self-regulation (e.g., Lewis et al., 2012). Furthermore, since self-awareness may lead to the development of a theory of mind (see the last section), a self-aware and “mentalizing” robot could better cooperate with humans and other AI agents. Bigman and Gray (2018) suggest that increasing elements of robot self-awareness as the theory of mind, situation awareness, intention, free will, could serve as a foundation for increasing human trust in robot autonomy because humans tend to judge the role of these and other perceived mental faculties as necessary in autonomy.

## Existing Approaches to Self-Awareness in Robots

McCarthy introduced the problem of robot self-awareness in a seminal paper (McCarthy, 2002), where he proposed a version of the Situation Calculus dealing with self-reflection, to make robot aware of their mental states.

The book by McDermott (2001) on “Mind and Mechanisms” is devoted to discussion of the computational theory of awareness, with similarities with the previous proposal by McCarthy. Chella and Manzotti (2007) and Holland (2003) collected the initial attempts at computational models of robot awareness and self-awareness. Reggia (2013) compiled an almost up to date review of the literature in the field. Scheutz (2014) reviewed, and discussed the relationships between robot awareness and artificial emotions.

Among the essential works concerning robot awareness, we consider the cognitive architectures based on the global workspace theory (Baars, 1997) as the LIDA architecture proposed by (Franklin, 2003; Franklin et al., 2014) and the architecture introduced by Shanahan (2005, 2006). Kuipers (2008) discussed a model of awareness based on learning and sensorimotor interaction in an autonomous robot.

Novianto and Williams (2009) put forth an attentive self-modifying framework (ASMO), arguing that some robot systems: (1) employ some aspects of self-awareness (e.g., recognition, perception), (2) ignore the role of attention, and (3) are too resource-intensive. Novianto (2014) updated ASMO, adding that a self-aware system attends to its internal states using a “black-box design” where each process is separate: (1) an attention mechanism mediates competition, (2) an emotion mechanism biases the amount of attention demanded by resources, and (3) a learning mechanism adapts attention to focus on improving performance.

Lewis et al. (2012) note that the involvement of collective (not singular) processes in self-awareness is potentially crucial for developing autonomous, adaptive AI that can balance tradeoffs between resources and goals. On the other hand, Habib et al. (2019) provide evidence that public, and private self-awareness processes (as one self-awareness node) can be used to balance trade-offs such as environment variation and system goals (corresponding transmission losses), respectively, via channel-hopping, in a self-aware self-redesign framework for wireless sensor networks.

Gorbenko et al. (2012) used a genetic algorithm (exons and introns) on their Robot Kuzma-II, defining robot internal states as non-humanoid states (i.e., robot control system, computing resources). Exons directly configure the system, and introns contain a meta-account of ongoing systems evolution. Monitoring these states triggers autonomous adaptation based on how well the robot's module recognizes incoming information. If the robot's modules provide low-quality recognition, then neural networks are used to generate a new module to improve identification and detection. The neural networks are also used to create simpler modules if incoming information is too dense.

Floridi (2005) proposed the knowledge game, a test for self-consciousness in agents based on the puzzle of three wise-man. There are three agents, and each agent receives one pill from a group of file pills, made by three innocuous and two dangerous pills. Now, according to Floridi, an agent may know its pill if the agent satisfies structural requirements for self-consciousness. Bringsjord et al. (2015) proposed a set of theoretical axioms for self-consciousness based on higher-order logics and a robot implementation of the axioms. They presented a robot effectively able to satisfy the Floridi test by interacting online with a human tester.

Design for robots involving self-awareness is, however, at the early stages (Chella et al., 2019). Many of these designs are based on working memory, reasoning, a theory of mind, socio-emotional intelligence, goals, experiences over development, and more (Chella et al., 2019). Cognitive architectures continue to integrate these ideas into a workable whole. For example, recently, Balkenius et al. (2018) architecture includes object permanence (remembering that a non-visible object still exists) and episodic memory (memories of one's life episodes), with mechanisms of sensation and perception running independently of sensory input to make room for planning and “daydreaming.”

Kinouchi and Mackin (2018) suggest a two-level architectural design: (1) awareness and habitual behavior, and (2) general goal-directed behavior, while Van Der Velde (2018) proposes that continuous cognitive access is controlled by “*in situ*” representations (e.g., open-ended questions/answers). Ye et al. (2018) offer a thorough review of AI cognitive architectures over 20 years, highlighting the need to bridge the gap between architectures based on problem-solving (engineering influence) and cognition (psychology) by theorizing and testing a varying range of functions across levels or phases of cognition, leading to hybrid designs.

Further theoretical work is being done to investigate how attention to the self may be vital in integrating other self-awareness processes (see, e.g., Graziano, 2017), and architectures continue to play a crucial role (Chella et al., 2018, 2019) in this respect. We agree that architecturally, attention to the self is essential for self-awareness, but we add that inner speech, at least in humans, is a primary tool for facilitating higher-order self-awareness and the many processes involved, such as memory, attention, reflection, social feedback, evaluation, and others presented earlier.

## Our Approach: Inner Speech

### Overview

When people talk to themselves in silence, they are engaging in “inner speech” (Alderson-Day and Fernyhough, 2015). Talking to oneself out loud (as well as in silence) is called “self-talk” (Hardy, 2006). Some synonyms of inner speech are “self-statements,” “phonological loop,” “internal dialogue,” “self-directed,” and “verbal thought” “inner speaking,” “subvocal,” “acommunicative,” or “covert speech” (Hurlburt et al., 2013). “Private speech” refers explicitly to self-directed speech emitted out loud by young children in social situations (Winsler et al., 2009).

Inner speech, seen as an instrument of thought, is compatible with the Language of Thought Hypothesis (LOTH) introduced by Fodor more than 40 years ago (Rescorla, 2019). LOTH suggests that thoughts possess a “language-like” or compositional structure (“mentalese”) with a syntax. Simple concepts combine in organized ways according to rules of grammar (like in natural language) to create thoughts; thinking takes place in a language of thought where thoughts are expressed as a system of representations embedded in a linguistic or semantic structure. In our view, inner speech represents a critical dimension of LOTH because of its inherent syntactic quality.

It is important not to confuse inner speech with other known inner experiences (Morin et al., 2019). Any *non-verbal* mental experiences, such as physical sensations, pure emotions, mental images, and unsymbolized thinking (“pure” thinking without the support of symbols), are *not* inner speech instances. Inner speech can take many forms, such as condensed (few words) or expanded (full sentences), and monolog (using “I”) or dialogue (asking questions and answering them using both “I” and “you”).

Inner speech is measured or manipulated with self-report scales, thought sampling and listing techniques, articulatory suppression, private speech recordings, electromyographic recordings of tongue movements (Morin, 2012; for a complete list of measures see Morin, 2018). Using these techniques has led to the identification of crucial functions served by inner speech, such as self-regulation (e.g., planning and problem-solving), language functions like writing and reading, remembering the goals of action, task-switching performances, the Theory-of-Mind, rehearsing person-to-person encounters, and self-awareness (Morin, 2018).

The inner speech represents an important cognitive tool beneficial to daily human functioning. However, it can lead to or maintain psychological disorders (Beazley et al., 2001), such as insomnia, boulimia/anorexia, agoraphobia, social anxiety, compulsive gambling, male sexual dysfunction, and more. Furthermore, inner speech use correlates with rumination discussed earlier (Nalborczyk et al., 2017). Although it remains unclear how to do so in humans exactly, dysfunctional inner speech in robots will most likely be kept in check through the cognitive architecture discussed later in this paper.

Inner speech emerges out of one's social environment, where first comes social speech, followed by private speech, and finally, inner speech (Vygotsky, 1962). In other words, inner speech represents the outcome of a developmental process during which linguistic interactions, such as between a caregiver and a child, are internalized. The linguistically mediated explanation to solve a task becomes an internalized conversation with the self. During the interview, the child is engaged in the same or similar cognitive tasks. The frequency of children's private speech peaks at 3–4 years, diminishes at 6–7 years, and gradually disappears and becomes mostly internalized by age 10 (Alderson-Day and Fernyhough, 2015). Nevertheless, many adults do occasionally engage in external speech when they are alone, for self-regulatory purposes, search and spatial navigation, for concentration, and emotional expression, and control (Duncan and Cheyne, 1999). Therefore, it is even more conceivable that a humanoid robot can relate to others by talking out loud.

Baddeley (1992) discusses the roles of rehearsal and working memory, where different modules in the working memory are responsible for the rehearsal of inner speech. The “central executive” controls the whole process; the “phonological loop” deals with spoken data, and the “visuospatial sketchpad” manipulates information in a visual or spatial form. The phonological loop is composed of the “phonological store” for speech perception, which keeps data in a speech-based way for a short time (1–2 s), and of the “articulatory control process” for speech production, that rehearses and stores information in the verbal form from the phonological store.

Neuropsychological reports of the brain-damaged patients and data gathered using the brain imaging techniques suggest that the left inferior frontal gyrus (LIFG) constitutes a critical cortical area involved in inner speech production (Geva et al., 2011). Additional brain areas associated with inner speech use are the supplementary motor area, the Wernicke's area, the insula, the right posterior cerebellar cortex, and the left superior parietal lobe (Perrone-Bertolotti et al., 2014).

To summarize: inner speech plays a central role in our daily lives. A person thinks over her perspectives, mental states, external events, emotions by producing thoughts in the form of sentences. Talking to herself allows the person to pay attention to the internal, and external resources, to retrieve learned facts, to learn and store new information, to control, and regulate her behavior, and, usually, to simplify otherwise demanding cognitive processes (Alderson-Day and Fernyhough, 2015). Inner speech allows the creation of the structure of the perception of the external world, and the self, by enabling high-level cognition, self-attention, self-control, and self-regulation.

### Inner Speech in Robots

Inner speech can be conceived as the back-propagation of produced sentences to an inner ear. A person then rehears the internal voice she delivers. Mirolli and Parisi (2006) report that talking to oneself to re-present information could have been the result of a pressure for the emergence of language, as shown by a simple neural network model of language acquisition where the linguistic module and sensory module are independent and feed-forward (imitation, mimicry), until a synaptic connection between the two modules occurs. Running this model results in the improved categorization of the world by agents in the simulation.

Steels (2003) argues that language re-entrance, defined as feeding output from a speech production system back as input to the subsystem that understands that speech, allows the refining of the syntax during linguistic interactions within populations of agents. Through computer simulations with grounded robots, Steels shows that the syntax becomes complete and more complex by processing the previously produced utterances by the same agent.

In the same line, Clowes and Morse (2005) discuss an artificial agent implemented employing a recurrent neural network where the output nodes correspond to words related to possible actions (e.g., “up,” “left,” “right,” “grab”). When the words are “re-used” by back-propagation of output to input nodes, then the agent achieves the task in a minor number of generations than in the control condition, where the words are not re-used. Clowes (2007) proposed a self-regulation model that links the inner speech to the role of attention and compared this model to Steels' (2003) re-entrance model. Clowes (2007) argues for a more activity-structuring, behavioral role of inner speech in modeling, claiming that checking grammatical correctness of prospective utterances alone is not sufficient to account for the role of inner speech.

Continuing with the argument that inner speech can potentially serve self-awareness processes (e.g., attention, regulation, reflection, etc.) efficiently, Arrabales (2012) proposes that inner speech may be considered as a “meta-management system” regulating or modulating other cognitive processes, as in the CERA-CRANIUM cognitive architecture. Recently, Oktar et al. (2020) proposed a textual and conceptual version of the mirror self-recognition task for chatbots that is comparable to the ideas already presented (language re-entry, re-use), where the chatbot's output is re-directed to its input. Of note (although only briefly discussed) is that (1) the authors do not equate self-recognition with self-awareness *per se*, (2) kinesthetic and visual matching (recognition) does not involve social understanding in this case, and (3) following self-recognition mechanisms, an inner speech mechanism should serve self-awareness, autonomy, and potentially theory of mind mechanisms (similar to self-awareness, sense of self, and society of mind in Steels, 2003).

### Inner Speech and Self-Awareness

Inner speech is crucially associated with self-awareness (Morin and Everett, 1990; Morin, 2005, 2018); thus, inner speech implementation in AI agents represents a promising avenue toward establishing some form of artificial self-awareness. The main argument is that the verbal cataloging of self-dimensions via inner speech makes it possible for a person to be fully cognizant of them and to integrate these characteristics into a self-concept gradually (Morin and Joshi, 1990).

The empirical evidence supporting a link between inner speech and self-referential activities is summarized in Table 1 (for a detailed presentation, see Morin, 2018).

**Table 1:** Summary of some evidence supporting the connection between inner speech and self-awareness.

**Evidence**	**Author(s)**
Several studies report significant positive correlations between measures of self-related constructs (including self-awareness) and inner speech.	E.g., (Brinthaupt et al., 2009)
Inner speech loss following brain injury leads to self-awareness deficits.	(Morin, 2009)
There is an increased activation of the LIFG observed during completion of many self-reflection tasks such as endorsement of personality traits, autobiography, and prospection.	(Morin and Hamper, 2012)
Inner speech facilitates awareness of mind-wandering episodes, cognitive performance, and other self-monitoring processes.	(Perrone-Bertolotti et al., 2014; Bastian et al., 2017)
Studies using thought-listing procedures report frequent inner speech about the self.	(Morin et al., 2018; Racy et al., 2019)

Specific mechanisms have been put forward to explain the nature of the link between inner speech and self-awareness (Morin, 1993, 1995, 2005, 2018). We present four possible mechanisms here.

(1) Inner speech reproduces social mechanisms leading to self-awareness. For example, people frequently comment on personal characteristics, and behaviors of others (e.g., “you are good looking,” “you are always late”); this, in essence, constitutes Cooley (1902) Looking-Glass Self Theory, where (mostly verbal) reflected appraisals allow people to learn about themselves from others' feedback. The self may *re-address* to itself appraisals from others by means of inner speech (e.g., “Indeed, I am good-looking”), thus cementing social feedback, as well as critically evaluate such appraisals (e.g., “I am *not* always late, for instance I was on time for my dental appointment last Wednesday”), thus correcting potentially biased feedback. Such an internalized process (via inner speech) is postulated to activate self-reflection and deepen self-knowledge (Morin and Joshi, 1990). Thus, an AI agent could catalog social feedback and correlate it with its database of self-knowledge, and then use speech to represent logical conclusions about itself.

(2) Self-awareness can be conceived as a problem-solving process where focusing on and learning about the self is the “problem” (e.g., “Who am I?” “How do I feel?” “What did I just do?”). The inner speech, then, is the cognitive tool used to solve that problem. Inner speech has been shown to facilitate problem-solving in general (Kendall and Hollon, 1981). This process can be applied to the self-as-a-problem, where inner speech helps the person to (i) define what the problem is (for example, “What did I do?”); (ii) determine the optimal approach to the problem (for example, “I will remember what happened and everything I did in detail”); (iii) generate problem-solving self-verbalizations (for example, “The first thing I did was X. Then Y happened, and I then said Z”); (iv) evaluative comments (for example, “Good! I'm getting somewhere!”); (v) directive notes (for example, “I don't need to take this into consideration as it is not pertinent”). All the above processes, by definition, represent self-awareness processes guided by the use of inner speech. In theory, a robot could represent itself to itself using the process described above, problem-solving about itself more effectively.

(3) An undeniable principle is that observation is possible only if there is a distance between the observer and the observed thing (Johnstone, 1970). Thus, following this principle, *self*-observation is possible only if there is a distance between the person and observable self-aspects. Expressing to oneself “I feel sad” produces a redundancy, because what was an emotion of sadness is now *re-presented* in words to the self. In place of only one thing, i.e., the pure emotion, now there are two elements: the emotion and its linguistic re-presentation. When a person just experiences the emotion (or anything else for that matter), she is too immersed in the experience to really perceive it. The verbal representation by the inner speech creates redundancy, which leads to a higher 'psychological' distance between that specific self-element (sadness), and the self. This distance, instigated by inner speech, facilitates self-observation and the acquisition of self-information. A robot agent could thus potentially use language to externalize self-observations and add these to its database of self-information.

(4) Verbal labeling of self-features, mental episodes, and behaviors makes it possible for the self to recruit a vast vocabulary about oneself to better perceive complex self-related information (Morin, 2005, 2018; St. Clair Gibson and Foster, 2007). One can verbalize to oneself, “I feel angry,” in which case all that one learns about oneself is that one… is angry. However, if one additionally says to oneself in inner speech, “I feel angry… actually, I also feel disappointed and possibly sad,” this likely will lead to a deeper understanding of what one is emotionally going through because of the use of supplementary adjectives. Therefore, people can tag their mental states using a large number of nuanced labels via inner speech—thus increasing self-knowledge. We argue that the same could be done in AI agents. BY cognitive architecture, robots could label their mental experiences and behaviors to represent and expand their self-knowledge database. In conclusion, the above analysis justifies the importance of implementing inner speech in robots to implant some form of self-awareness in their architecture.

## A Cognitive Architecture for Inner Speech Implementation in Robots

**Figure 1:**
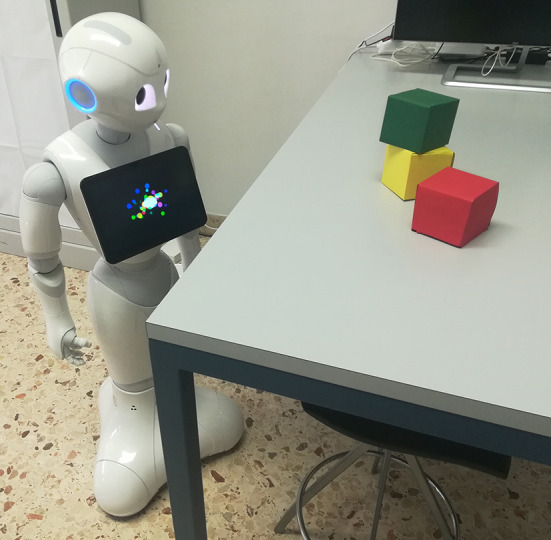
The Pepper robot employed for the experiments reported in the paper.

In this section, we describe a model of a cognitive architecture for robot self-awareness by considering cognitive processes and components of inner speech. It should be remarked that such operations are taken into account independently from the origin of linguistics abilities, which are supposedly acquired by a robot. In particular, we consider an implementation of the architecture mentioned above on a Pepper robot working in a laboratory setup (Figure 1).

**Figure 2:**
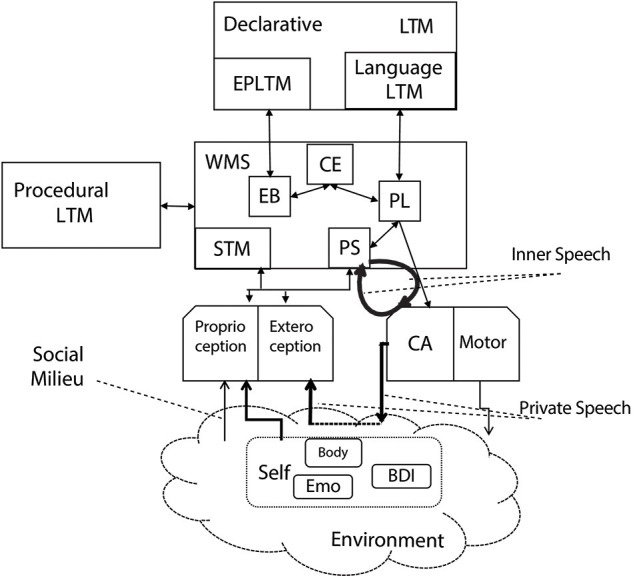
The cognitive architecture for inner and private speech.

Figure 2 shows the proposed cognitive architecture for inner speech. The architecture is based on the Standard Model of Mind proposed by Laird et al. (2017). The structure and processing are elaborated to integrate the components and the processes described in the inner speech theories previously discussed. A preliminary version of the architecture is reviewed in Chella and Pipitone (2020).

### Perception and Action

The perception module of the architecture receives perceptive input signals from the robot camera and proprioceptive signals from the inner robot sensors. The perception model of the proposed architecture includes the *proprioception* module related to the self-perception of emotions (Emo), belief, desires and intentions (BDI), and the robot body (Body), as well as the *exteroception* module which is related to the perception of the outside environment.

The proprioception module, according to Morin (2004), is also stimulated by the *social milieu* which, in the considered perspective, includes social interactions of the robot with others entities in the environment, as well as physical objects like mirrors and cameras and other robots or humans, by means of face-to-face interaction that fosters self-world differentiation.

The actuator module is decomposed into two sub-components: the *Covert Articulation* module (CA), and the *Motor* module (Motor). The Motor module is related to the actions the agent performs in the outside world. The Covert Articulation (CA) module rehearses the information from the Phonological Store (PS), which is the perceptual buffer for speech-based data and it is a sub-component of short-term memory (see below). Such a module acts as the inner speech heard by the phonological store by rounding information in a loop. In this way, inner speech links the covert articulation to the phonological store in a round loop.

### Memory System

The memory structure is divided into three types of memory: *short-term* memory (STM), *procedural* and *declarative* long-term memory (LTM), and *working* memory system (WMS). The short-term memory holds the sensory information from the environment in which the robot is immersed that was previously coded and integrated with information coming through perception. The information flow from perception to STM allows the storing the coded signals previously considered.

The information flow from the working memory to the perception module provides the ground for the generation of *expectations* on possible hypotheses. The flow from the phonological store to the proprioception module enables the *self-focus* modality, i.e., the generation of expectations concerning the robot itself.

The long-term memory holds the learned behaviors, the semantic knowledge, and in general the previous experience. *Declarative LTM* contains linguistic information in terms of lexicon and grammatical structures, i.e., the *Language LTM* memory. The declarative linguistic information is assumed acquired and represents the *grammar* of the robot. Moreover, *Episodic Long-Term Memory* (EBLTM) is the declarative long-term memory component that communicates with the *Episodic Buffer* (EB) within the working memory system, which acts as a “backup” store of long-term memory data.

**Figure 3:**
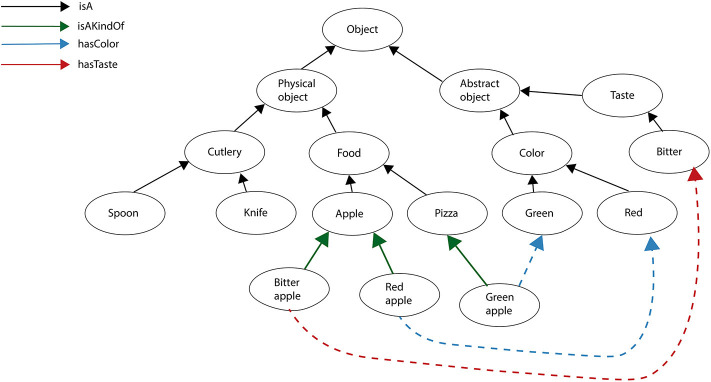
A fragment of the declarative LTM implemented by a semantic network.

Figure 3 reports a fragment of the *Declarative LTM* where a spoon and a knife are represented as pieces of cutlery, and an apple is represented as food. A bitter apple and a red apple are kinds of apple, A bitter apple has a bitter taste, and a red apple has a red color. Examples of *Language LTM* expressed in terms of the Fluid Construction Grammar formalism (Steels, 2003) may be found in Micelli et al. (2009).

The *Procedural* LTM contains, among others, the composition rules related to the linguistic structures for the production of sentences at different levels of complexity.

Finally, the working memory system contains task-specific information “chunks” and it streamlines them to cognitive processes during task execution step by step of the cognitive cycle. The working memory system deals with cognitive tasks such as mental arithmetic and problem-solving. The *Central Executive* (CE) sub-component manages and controls linguistic information in the rehearsal loop by integrating (i.e., combining) data from the phonological loop and also drawing on data held in long-term memory.

### The Cognitive Cycle at Work

The cognitive cycle of the architecture starts with the perception module that converts external signals in linguistic data and holds them into the phonological store. Thus, the symbolic form of the perceived object is produced by the covert articulator module of the robot. The cycle continues with the generation of new emerging symbolic forms from long-term and short-term memories. The sequence ends with the rehearsing of these new symbolic forms, which are further perceived by the robot. Then, the cognitive cycle restarts again.

**Figure 4:**
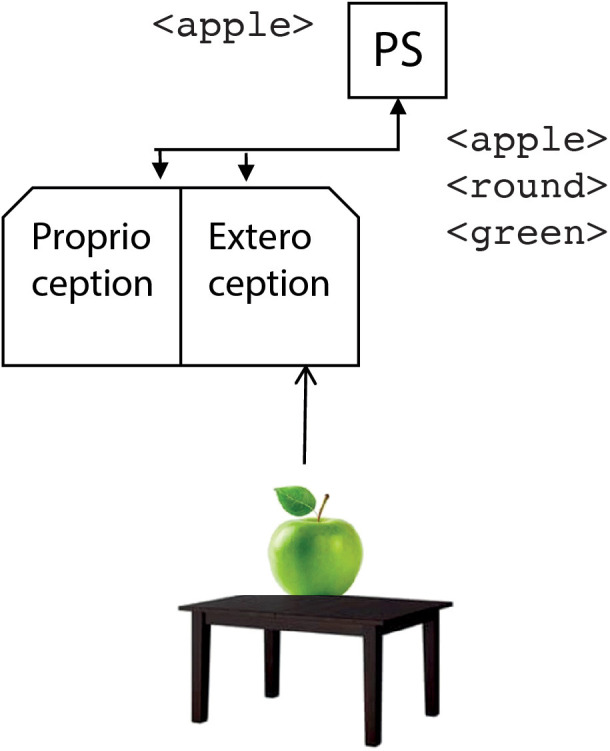
The operation of the perception module. It classifies the input signals by generating suitable symbolic labels that are sent to the phonological store (PS).

Let us consider a scenario with some fruits and pieces of cutlery on a table. In the beginning, the robot perceives an apple. Thus, the perception system generates the labels <apple>, <round>, <green> that are sent to the phonological store. The phonological store processes one of the words generated by the perception system; in our case, the word <apple> (Figure 4).

In the current system, the processing of words happens in a first-in-first-out queue: the <apple> is the first word generated by the perception system, and it is the first one to be processed by the phonological store.

It is to be remarked that the label arriving at the phonological store is the same as if someone from outside would pronounce the word “apple.” In this sense, the phonological store works as an inner ear. This is the entry point of the phonological loop.

**Figure 5:**
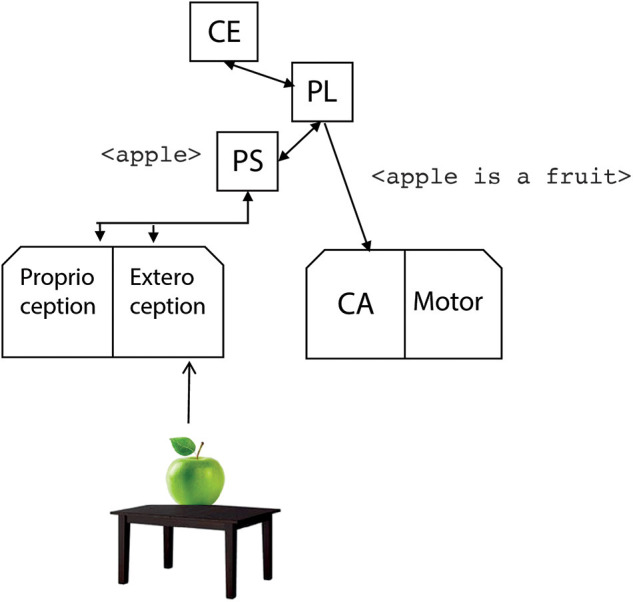
The phonological store receives in input the label <apple> generated by the perception module. Then, the central executive (CE) looks for information from the LTM and the phrase <apple is a fruit> is generated by the phonological loop (PL).

The central executive CE enters in action to process the input <apple> by querying the STM, the Procedural and Declarative LTM. As a result, the phrase <apple is a fruit> is generated thanks to the linguistic rules stored in the LTM and sent to the covert articulation module (Figure 5).

**Figure 6:**
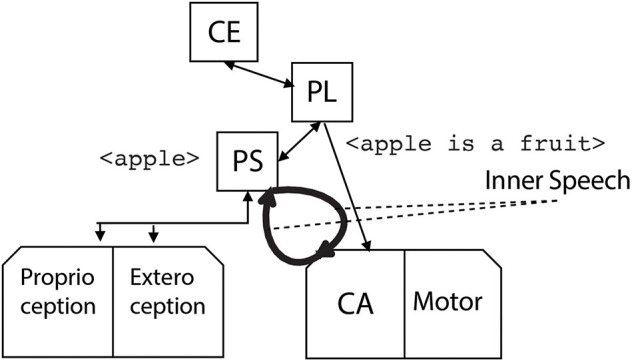
The robot internally rehearses the phrase <apple is a fruit> by the covert articulation module, thus generating the robot inner speech.

**Figure 7:**
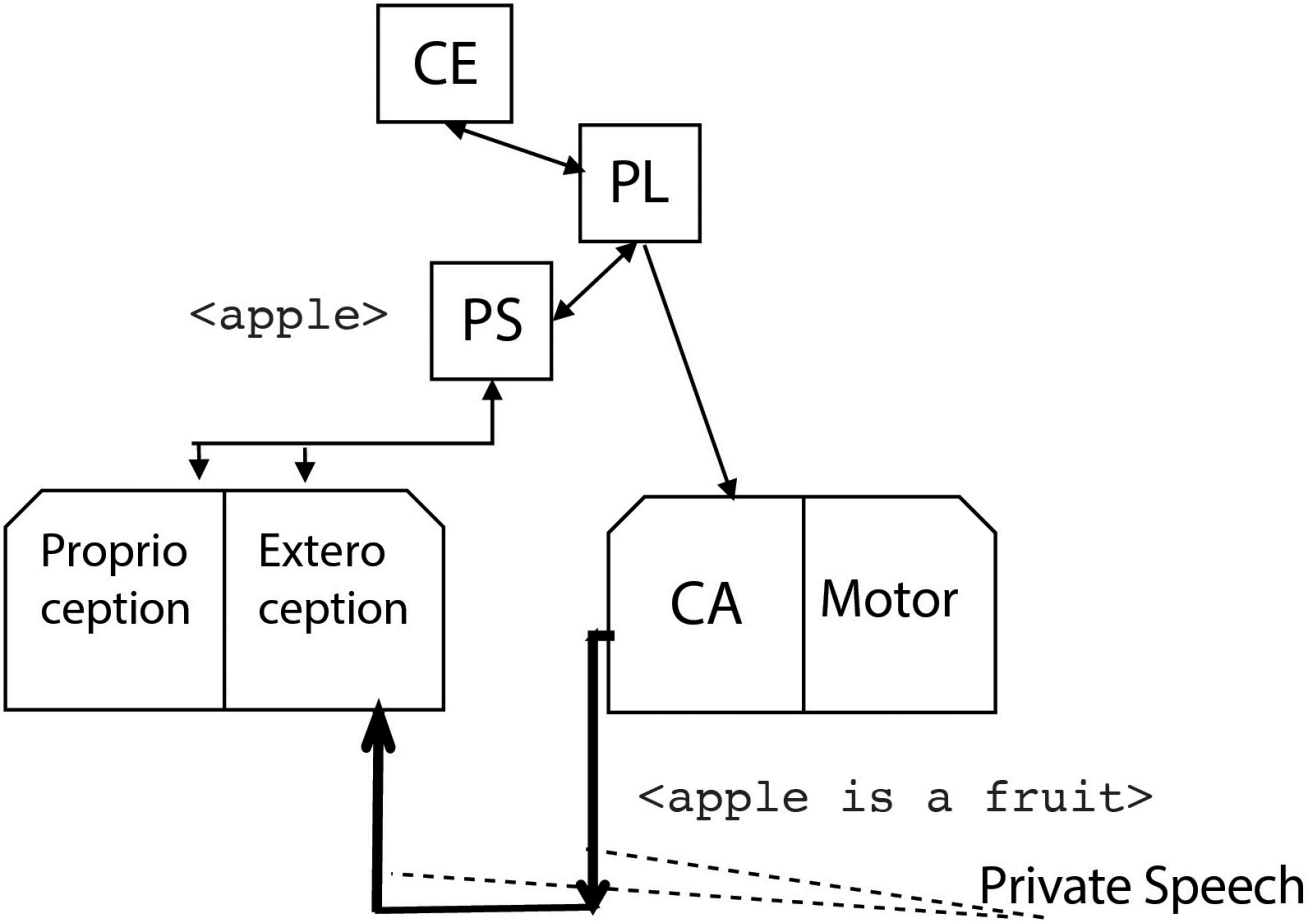
The robot externally rehearses the phrase <apple is a fruit> by the covert articulation module. The phrase is in turn perceived by the perception module, thus generating the robot private speech.

Now, the generated phrase reenters the phonological store as a new input of the phonological loop. Two ways are available for the reentering: the inner speech mode, where the phrase internally reenters the phonological store, without being externally audible (Figure 6), and the private speech mode, where the phrase is effectively generated by the covert articulation module so that it is a new input to the exteroception module (Figure 7).

**Figure 8:**
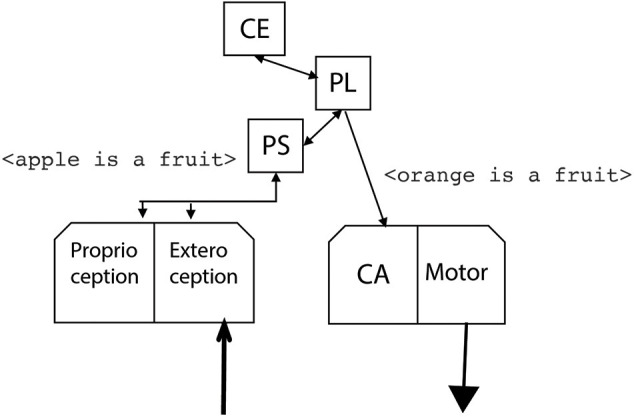
The robot associates the apple with the orange as they are both fruits and it generates the phrase <orange is a fruit> which in turn generates the expectation of an orange in the scene by the motor module.

The reentered phrase elicits again the central executive, which queries the procedural and declarative LTM. Now, oranges and apples belong to the same category of fruits, and then the central executive generates an expectation for orange in the scene. The result is the generated phrase <orange is a fruit> (Figure 8) as a result of behavioral rules stored in the Procedural LTM.

**Figure 9:**
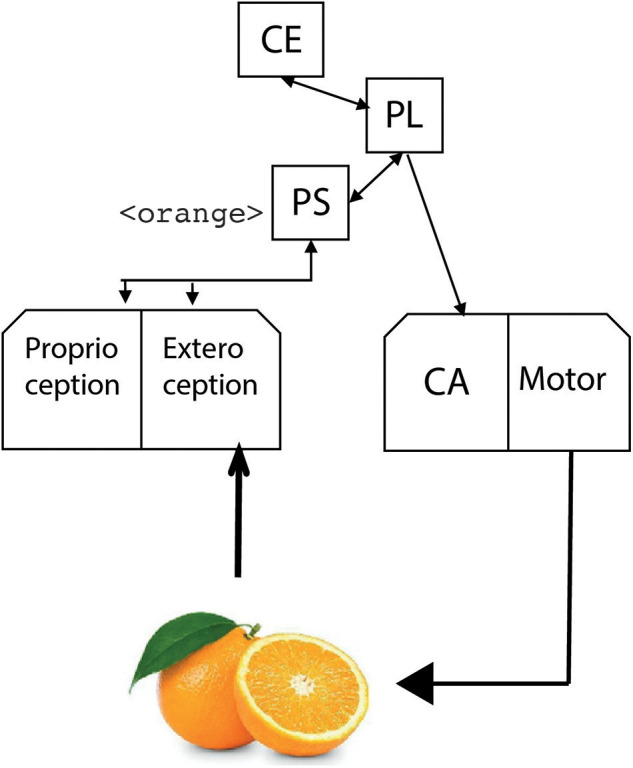
The expectation of an orange in the scene is satisfied by the perception module which generates the label <orange>.

The central executive then starts a search for oranges in the scene by controlling the motor module of the robot. The search is confirmed by the perception system, and the word <orange> is generated (Figure 9). Again, the phonological loop enters in action, this time generating the word <knife>, which is confirmed by the perception system.

The generation of language in the current system is based on the semantic network reported in Figure 3. The system generates and processes trigrams based on the predicates listed in the upright corner of the figure. For example: <apple isA food>, <red_apple isAKindOf apple>, <red_apple hasColor red>, <bitter_apple hasTaste bitter>.

The computational model takes into account two kinds of rules to generate expectations (Chella et al., 1997). On the one side, a rule makes expectations based on the structural information stored in the symbolic knowledge base of the LTM. An apple is a fruit, and then other fruits may be present in the scene. As soon as an object is recognized, then other objects belonging to the same class may be present, and so an expectation is generated. We call these expectations *linguistic*.

The linguistic expectations are hard coded in the current system. For example, if <object_x1> and <object_x2> are subclasses of <object_X> and there is an <object_x1>, then generate an expectation of <object_X>.

On the other side, expectations are also generated by purely associative mechanisms between objects. Suppose that the system learned that when there is fruit in the scene, then there is also usually some cutlery. The robot thus will learn to associate these two objects and to perform the related search when finding one of the two objects. Then, a <fruit>, generated by the speech recognition system or by the vision system, will be associated with the word <knife>. We call these expectations *associative*.

During a training phase, the system stores lists of diverse entities that are present at the same time in the scenario, as (<apple>, <knife>); (<pear, fork>); (<orange>, <spoon>), and so on. Then, each word is coded by a string of bits according to a sparse random code, and the previously listed training set is learned by an attractor neural network (see, e.g., Amit, 1988). This framework suited well in the described simplified scenario. Similar associative schemas are defined by Kosko (1988), Pollack (1990), Plate (1995). Thomson and Lebiere (2013) proposed a complex associative learning mechanism integrated into the ACT-R cognitive architecture.

Finally, in the described example, the inner/private speech of the robot is composed by the phrases: <apple>, <apple is a fruit>, <orange is a fruit>, <orange>, <knife>. It is an example of inner/private speech concerning an explorative task: the robot explores a scene employing linguistic and associative inferences. The expectations of the robot are made explicit through private robot speech. Again, it should be noticed that inner/private speech reenters the information generated by the architecture as a new input of the architecture itself.

Let us now consider a dynamic scene, for example, a person moving her arm toward the apple. In this case, when the robot recognizes the motion of the forearm, then it infers the presence of a moving upper arm. In this case, the system recognizes a situation of a moving arm as made up of the synchronous motion of the forearm and the upper arm. The resulting inner speech is: <forearm is moving>, <upper arm is moving>, <arm is moving>. We call this type of expectation *synchronic* because they refer to the synchronous situation of two moving entities at the same time.

The recognition of a specific situation in the scene could elicit the inference of change in the arrangement of entities in the scene. We call this kind of expectation *diachronic* in the sense that it involves a sequence of situations. Diachronic inferences can be related to the link existing between a situation perceived as the precondition of action, and the corresponding situation expected as the effect of the action itself. In this way, diachronic inferences prefigure the situation resulting in the outcome of an action (see also Chella et al., 2000).

Let us consider the case of the moving arm grasping an apple: in this case, the previous situation of the moving arm and the apple on the table evolves in a new situation where the arm now holds the apple. The grasp action will be then recognized. The generated inner/private speech is the following: <forearm is moving>, <upper arm is moving>, <arm is moving>, <arm holds apple>, <grasp apple>.

On the one side, expectations are related to the structural information stored in the symbolic knowledge base, as in the previous example of the action of grasping. We call these expectations linguistic, as in the static case. As soon as a situation is recognized, and the situation is the precondition of action, the symbolic description elicits the expectation of the effect situation, and then the system recognizes the action itself.

On the other side, expectations are also related to purely associative mechanisms between situations. Suppose that the system learned that when there is a grasp action, then the action is typically followed by a move action. The system could learn to associate these two subsequent actions. We call these inferences associative, as in the static case.

The robot will thus explore a dynamic scene driven by linguistic and associative expectations. Even in this case, the sequence of robot expectations is made explicit employing the robot's inner/private speech, which has the role of reentering the emerging expectations and eliciting new ones.

In the two previous scenarios, the robot passively observes and describes static and dynamic scenes. The third scenario is a natural extension of the previous one, where the robot is able to observe itself and explain its actions (see also Chella et al., 2008). Let us consider the case where a robot recognizes the apple, and it moves its arm to grasp the apple. The movements of the robot arm are planned and controlled by low-level robot control routines. Then, the robot monitors the movements of its arm by its camera, and its motion sensors to describe its actions. In this case, the inner/private speech generated is similar to the previous one: <my forearm is moving>, <my upper arm is moving>, <my arm is moving>, <my arm holds apple>, <I grasp apple>. The difference concerns the fact that the robot recognizes that the moving arm is its arm by the examination of the proprioceptive and perceptive sensors, i.e., by the motor sensors of the arm and the camera. Then, the robot is able to generate expectations about itself by putting into action the self-focus modality. As a result, the robot performs a simple form of self-awareness: the inner/private speech concerns its actions.

In summary, the robot, thanks to the reentering of its inner/private speech, is able to describe static and dynamic scenes in front of it to empower the robot situational awareness. The robot is also able to represent itself by observing and describing its actions to enable a simple form of self-awareness.

## Discussion

The focus of research is investigating the role of inner and private speech in the robot task of the exploration of a scene. To the knowledge of the authors, no other robot system employed inner or private speech, as described in the previous sections. The implemented framework is based on a simplified setup to focus the study on robot inner speech by avoiding the well-known problems related to vision, action, and language.

The current implemented system is tailored to the described simplified scenario of fruits, and cutlery on a table. The employed vision system is not able to deal with ambiguities. An extended robot vision system able to deal with static scenes and dynamic scenes is described in Chella et al. (1998, 2003). The system is able to learn from examples (Chella et al., 2006) and to deal with ambiguities (Chella et al., 2010). The integration of the extended vision system with inner and private speech mechanisms will be the object of future investigations.

While our approach favors inner and private speech in an attempt to produce a simple form of self-awareness in AI agents, other factors also need to be examined for the eventual development of full-blown human-like self-awareness. As alluded to before, Morin (2004, 2011) suggests three sources of self-awareness: (i) the self; (ii) the physical world; and (iii) the social environment. Although the proposed cognitive architecture offered above does include some simplified elements only within these sources, additional sub-processes should be taken into account. Below we discuss those sub-processes that arguably seem most important: social comparison, mental imagery, future-oriented thinking, and Theory-of-Mind.

Social comparison represents the process by which people evaluate themselves by comparing themselves to others to learn about the self (Festinger, 1954). For example, John might observe that most of his colleagues leave work earlier than him, or that many are thinner than he is, leading him to conclude “I am a hardworking person” or “I am overweight.” As this illustration suggests, inner speech is most likely activated at one point or another during the social comparison. This process is far from perfect because of various self-protective and self-enhancement biases that it entails. Individuals may interpret, distort, or ignore information gathered by social comparison to perceive themselves more positively (e.g., Eichstaedt et al., 2002). For instance, they may opt to engage in upward comparisons (comparing themselves to someone better off) or downward comparisons (comparing themselves to someone worse off), or avoid comparisons as a function of their self-enhancement needs. Despite these limitations, social comparison certainly constitutes an authoritative source of self-information and self-knowledge. Computers, as well as some other AI entities, are already connected via the internet and thus, theoretically, could “see” others and compare themselves to them.

Mental imagery constitutes a visual experience in the absence of the visual stimulus from the outside environment (Morris and Hampson, 1983). Because mental imagery in humans leads to the development of autoscopic imagery (i.e., images of the self, especially one's face and body; Morin and DeBlois, 1989), it plays a potentially important role in self-awareness. Although empirical evidence is sparse, Turner et al. (1978) observed that highly self-aware people report using the imagery to engage in introspection. To illustrate, one can mentally generate (or replay) scenes in which the self is an actor (e.g., relaxing at the beach). Self-aspects (e.g., an emotion of contentment) can be inferred from what the actor is mentally seen doing (e.g., smiling). Like inner speech, mental imagery can internally reproduce and expand social mechanisms involved in self-awareness, such as the possibility of seeing oneself (literally) as one is seen by others. From a self-awareness perspective, robots would certainly benefit from mental imagery, although it remains currently unclear how to implement such a process.

Future-oriented thinking represents the capacity to think about events that are relevant to the future of the agent (Szpunar, 2010; Schacter et al., 2017). It rests on the ability to think about one's past (episodic memory, autobiography), as personal memories provide the building blocks from which episodic future thoughts are created. The contents of episodic memory are sampled and recombined in different ways, leading to the construction of mental representations of future scenarios (Tulving, 1985). As an example, in imagining the personal experience of moving, one can rely on remembering one's previous moves—how it felt, how long it took, how much money it cost, etc. Four types of future-oriented thinking have been put forward (Szpunar et al., 2016; Schacter et al., 2017): (1) *simulation*, or the creation of a precise mental representation of one's future, (2) *prediction*, or the estimation of the likelihood that a future outcome will occur, (3) *intention*, or goal setting, and (4) *planning*, or the steps needed to attain a goal. It would be advantageous to endow a robot with future-oriented thoughts. Since the cognitive architecture presented earlier includes episodic long-term memory, it already possesses the fundamental ingredient for such thoughts to take place.

The Theory of Mind is defined as the ability to attribute mental states as intentions, goals, feelings, desires, beliefs, thoughts, to the others (Gallagher and Frith, 2003). It allows human beings (and arguably some non-human animals—see Gallup, 1968, 1997) to predict others' behavior, to help and cooperate, to avoid, or to deceive the others, and to detect cheating (Malle, 2002; Brüne and Brüne-Cohrs, 2006). As such, organisms capable of Theory-of-Mind gain a major adaptative and survival advantage. According to the Simulation Theory, people internally simulate what others might be experiencing inside by imagining the sort of experiences they might have when in a similar situation (Focquaert et al., 2008). Thus, according to this view, self-awareness represents a prerequisite to Theory-of-Mind. It is conceivable that machines made self-aware via inner speech implementation could engage in Theory-of-Mind, especially since the former most likely is implicated in the latter (Fernyhough and Meins, 2009). However, the precise operations required for the development of artificial Theory of Mind remain elusive at present—but see Vinanzi et al. (2019) and Winfield (2018), among others.

## Conclusion

We discussed self-awareness and inner speech in humans and AI agents, followed by an initial proposal of a cognitive architecture for inner speech implementation in a robot. Although several authors have put models of self-awareness development in robots, our approach focuses on inner speech deployment as a privileged method for reaching this elusive goal because of the strong ties that exist between self-awareness and inner speech. The suggested architecture consists of an integration of vital cognitive elements following Laird et al. (2017) and includes theoretical insights offered by Baddeley (1992), Morin (2004), Steels (2003), Clowes (2007), and others. Cognitive operations such as short-term memory, working memory, procedural and declarative memory, and covert articulation represent established factors in conscious human experience. We anticipate that once activated in the cognitive cycle described earlier, these components (as well as several others) will replicate self-awareness via inner speech in robots.

One effort will be to test the establishment of self-awareness in AI agents empirically. Our approach offers the advantage that robots' inner speech will be audible to an external observer, making it possible to detect introspective and self-regulatory utterances. Measures and assessment of the level of trust in human-robot interaction involving vs. not involving robot inner speech will be the object of further investigations.

## Data Availability Statement

The datasets generated for this study are available on request to the corresponding author.

## Ethics Statement

Written informed consent from the participants' legal guardian/next of kin was obtained for the publication of any potentially identifiable images or data included in this article.

## Author Contributions

AC and AM contributed conception and design of the study. AP contributed the proposed computational architecture and performed robot experiments. FR and AM contributed to the psychological aspects of the study. All authors contributed to manuscript revision, read and approved the submitted version.

### Conflict of Interest

The authors declare that the research was conducted in the absence of any commercial or financial relationships that could be construed as a potential conflict of interest.
